# The Psychosocial Impact of the Decision to Undergo Risk-Reducing Salpingo-Oophorectomy Surgery in *BRCA* Mutation Carriers and the Role of Physician-Patient Communication

**DOI:** 10.3390/curroncol30020185

**Published:** 2023-02-17

**Authors:** Ana C. Alves-Nogueira, Daniela Melo, Carlos Carona, Margarida Figueiredo-Dias

**Affiliations:** 1 Center for Research in Neuropsychology and Cognitive and Behavioral Intervention, Faculty of Psychology and Educational Sciences, University of Coimbra, Rua do Colégio Novo, s/n, 3000-115 Coimbra, Portugal; 2 Gynecology Clinic, Faculty of Medicine, University of Coimbra, 3004-531 Coimbra, Portugal; 3 Gynecology Department, Coimbra University Hospital Centre, 3004-561 Coimbra, Portugal

**Keywords:** risk-reducing salpingo-oophorectomy (RRSO), *BRCA* mutation, ovarian cancer, breast cancer, shared decision-making, physician-patient communication (PPC), psychosocial adjustment

## Abstract

Risk-reducing bilateral salpingo-oophorectomy (RRSO) is an effective prophylactic surgery provided to premenopausal women carrying *BRCA1* or *BRCA2* mutations and presenting an increased risk of developing breast or ovarian cancer. This procedure is related to physiological, sexual, and psychosocial distress, which altogether increase uncertainty and complexity in the clinical decision-making process and post-surgery adaptation. Physician-patient communication (PPC) has been pointed out as a determinant factor in the decision-making to undergo RRSO, and the subsequent adjustment of women. However, studies examining the psychosocial impact of the decision-making process have been scarce and often lack clear theoretical frameworks. While the role of PPC in such processes has been highlighted in a few qualitative studies, there is a paucity of quantitative research addressing this question. Therefore, this narrative review, conducted using a multidisciplinary approach, was planned to: (1) present an updated medical background for RRSO; (2) analyze the psychosocial impact of the decision-making process within a theoretical framework of the Health Belief Model; and (3) discuss the role of PPC in such a decision-making process and in post-surgery. The collected research also enabled the recommendation of some additions to the existing clinical guidelines and the outlining of future research directions.

## 1. Introduction

Hereditary breast and/or ovarian cancer are associated with specific genetic variants that greatly increase the lifetime risk of developing these conditions [1,2]. For instance, some rare gene mutations correlate with breast and/or ovarian cancer predisposition syndromes [2,3,4,5]. However, the most common and well-known examples are the pathogenic *BRCA1* and *BRCA2* genomic variants, present in 1 to 300–500 women and responsible for 5–10% of breast cancers, and 10–15% of ovarian cancers [3,4,6,7,8,9].

As long as *BRCA1* and *BRCA2* mutations are involved, risk-reducing strategies should be implemented to prevent the risk of cancer, such as risk-reducing salpingo-oophorectomy (RRSO). RRSO is an effective risk-reducing surgery recommended to high-risk premenopausal healthy women carrying these mutations, ideally after the age of 35 or immediately after childbearing is complete [10,11]. It includes the surgical removal of the fallopian tubes and ovaries bilaterally and has proved its effectiveness in preventing ovarian cancer in *BRCA1/2* carriers [1,6]. Nevertheless, the removal of apparently healthy organs may have serious physiological, sexual, and psychological consequences and does not ensure the elimination of future cancer risk.

The decision-making process to undergo RRSO is highly challenging for women and depends on multiple variables, such as personal appraisals, medical history, and psychological functioning [12]. Several qualitative studies have analyzed how these variables interact and contribute to the decision-making process (e.g., [13,14]). Even though a solid theoretical framework suggests the synthesis of potential interrelations between determinants, processes, and outcomes, ultimately guiding research and clinical intervention in shared decision-making processes [15], this has been systematically lacking in the aforementioned research.

Additionally, the decision to undergo RRSO is expected to be shared between the woman and her physician/oncologist. In this sense, physician-patient communication (PPC) has been acknowledged as a crucial factor in patient-centered care and associated with positive effects over healthcare outcomes (e.g., [16,17,18]). PPC also appears to be distinctively linked to women’s adaptation outcomes after RRSO [13,19,20,21].

In this context, a narrative review [22,23] was conducted employing a multidisciplinary approach (i.e., medicine and psychology) in order to analyze and integrate (i) the psychosocial impact of the decision-making process to undergo RRSO in women carrying *BRCA* mutations, and (ii) the role of PPC during this process and in post-surgery. Specifically, this narrative review was planned to: (1) present an updated medical background for RRSO, namely the genomic framework that leads its medical recommendation, among other possible alternative prophylactic measures, and its physiological consequences; (2) analyze the psychosocial impact of the decision-making process, within a theoretical framework of the Health Belief Model; and (3) discuss the role of PPC in women’s decision-making processes and post-surgery adaptation outcomes.

## 2. Clinical Background for the Recommendation of RRSO

### 2.1. Genomic Susceptibility to Ovarian/Breast Cancer

Some rare moderately penetrant genes confer an increased risk of developing hereditary breast and/or ovarian cancer, such as *MLH1*, *MSH2* or *STK11* [2,3,4,5]. Table 1 summarizes the risk rates for developing breast and/or ovarian cancer for each genomic pathogenic variant [1,3,4,5,6,8,9,24,25,26,27,28,29,30,31,32].

Despite the risk conferred by these rare pathogenic variants, research is still scarce on this topic, preventing the establishment of clinical management protocols for most of them [5].

It is noteworthy that most cases of hereditary breast and/or ovarian cancers are associated with germline mutations in *BRCA1* and *BRCA2* genes [8]. *BRCA1* and *BRCA2* mutations are responsible for 5–10% of breast cancers and 10–15% of ovarian cancers [3,4,6,9]. Besides the high cumulative risk at the age of 70–80, women carrying these mutations may already present an increased risk up to 10% for developing ovarian cancer by the age of 50 [4]. The 5-, 10-, and 15-year cumulative risk for *BRCA1* carriers is 13.7%, 23.8%, and 36.1%, respectively; and for *BRCA2* carriers is 12.0%, 18.7% and 28.5%, respectively [33].

Given the lifetime and cumulative risks of cancer in *BRCA* germline mutation carriers, after genetic testing disclosure, risk-reducing strategies are usually recommended by clinical practice guidelines [2,7,10,30].

### 2.2. Risk-Reducing Measures and RRSO

Various strategies can be used to reduce cancer risk, morbidity, and mortality in women with an increased risk of hereditary breast and ovarian cancer [5], within a multidisciplinary approach [3]. Table 2 represents a summary of risk-reducing measures recommended by clinical guidelines for *BRCA* mutation carriers [1,2,3,6,7,8,10,27].

Regarding the screening methods, mammography seems to be the only effective imaging strategy in reducing breast cancer mortality. However, it presents lower detection sensitivity in *BRCA* mutation carriers in comparison to the general population, particularly in women under 40 years old and carrying a *BRCA1* variant. It is noteworthy that breast MRI is reported as the most sensitive screening exam for *BRCA* mutation carriers. Still, data are missing about the effectiveness of this strategy in reducing long-term mortality in these women [34]. On the other hand, compared to breast cancer screening, ovarian cancer screening methods are largely ineffective, with no reported benefit in reducing ovarian cancer mortality [3,35]. Specifically, in a 2007 observational, follow-up study in the general population, annual screening with combined CA125 and transvaginal ultrasound failed to detect early-stage cancer. In fact, there were women diagnosed with stage III/IV cancers, while having a normal screening 3 to 10 months before diagnosis [6]. Moreover, the large multicenter, prospective phase II, UK Familial Ovarian Cancer Screening Study screened 348 high-risk women using an algorithm that combined CA125 results every 4 months and annual transvaginal ultrasound. Similarly, the MODENA study prospectively screened 661 women with pathogenic *BRCA1/2* variants using CA125 and transvaginal ultrasound every 6 months. Even though screening in both studies was associated with diagnosis of early-stage disease (stage I or II), it failed to reduce ovarian cancer mortality [1,7,8]. Therefore, clinical guidelines recommend that high-risk women should be aware of the limitations of solely using ovarian cancer screening as a prevention strategy, and possibly consider other more effective prophylactic measures [1,7,8,30].

The evidence of the effect of oral contraception on breast cancer risk among *BRCA1/2* mutation carriers is still controversial. Although a few case-control studies have reported a modest increase in breast cancer risk [8], at least two meta-analyses showed no increased risk of breast cancer in women with *BRCA1/2* mutation taking oral contraception [1,3,6,27]. Additionally, oral contraceptives can reduce the risk of ovarian cancer by 40–50% [1,2,3,6,27] in *BRCA* carriers, and the benefit is greater the longer the treatment [1,2,8]. A meta-analysis published in 2013 included 14 studies and showed a 42% reduction in the risk of ovarian cancer associated with combined hormone contraceptive use in women with *BRCA1/2* variants [1]. However, there is a lack of both randomized and controlled trials to determine the effectiveness of this prophylactic measure [27].

The use of tamoxifen and aromatase inhibitors (AI) as chemoprevention strategies has shown efficacy in reducing the risk of invasive breast cancer in high-risk postmenopausal women. However, there are no prospective studies evaluating the risk-reduction effect of tamoxifen in women with *BRCA* mutations. Available data suggest a benefit for individuals carrying a *BRCA2* mutation but possibly not in *BRCA1* mutation carriers. Available retrospective data suggest that AI can have a real benefit in reducing breast cancer in *BRCA1/2* carriers [2,10].

Women carrying *BRCA1* and *2* mutations have a breast cancer risk reduction of at least 90% when undergoing risk-reducing mastectomy (RRM) [2,3,8,27,32,35,36]. This risk reduction also translates in a 90% decrease in cancer-related mortality [35]. In addition, RRM is associated with low rates of postoperative complications and reduced rates of surgery-related mortality, even though the risk of developing breast cancer is not fully eliminated [8]. Nonetheless, NCCN guidelines recommend the discussion of this option with women carrying *BRCA1* and *2* mutations [6,7], since RRM has a negative impact on body image and sexuality and is frequently associated with long-term physical symptoms [10]. It is worth noting that risk-reducing mastectomy has no prophylactic effect regarding the risk of developing ovarian cancer, thus highlighting the need to consider a more effective alternative targeting both types of cancer.

As for RRSO, surgical removal of both ovaries and fallopian tubes in premenopausal women significantly reduces the levels of circulating hormones, which may lead to a reduced risk of developing estrogen-dependent breast cancer. Some authors continue to argue that RRSO leads to a 40–70% risk reduction of developing breast cancer [1,2,3,4,6,24,32,33,36,37]. However, hereditary breast cancer is often triple-negative, so hormonal mechanisms alone are not enough to cause breast cancer [38]. In addition, a prospective multicenter cohort study reported that RRSO significantly reduced the risk of breast cancer for *BRCA2* pathogenic variant carriers but not for *BRCA1* carriers [36]. In turn, when performed in premenopausal women carrying *BRCA1/2* mutations, RRSO reduced the risk of ovarian cancer by 80–96% [1,2,4,6,7,8,24,26,27], as well as the mortality rate associated with ovarian cancer by 94% [24]. Specifically, a 2018 Cochrane review compared 2936 women with pathogenic *BRCA1/2* variants undergoing RRSO with 5151 women not undergoing this surgery but following other surveillance measures. This study demonstrated that undergoing RRSO decreased both ovarian cancer (HR 0.06; 95% CI (0.02–0.17)) and breast cancer mortality (HR 0.58; 95% CI (0.39–0.88)) [1]. Moreover, in a large 2016 prospective study, RRSO was effective in breast cancer prevention in *BRCA2* mutation carriers (age-adjusted HR 0.18; 95% CI (0.05–0.63)). However, in the same study, an effective breast cancer prevention through RRSO for *BRCA1* mutation carriers was not evident (age-adjusted HR 0.79; 95% CI (0.55–1.13)) [7].

Despite the evidence for the effectiveness of RRSO, the risk of developing peritoneal cancer after undergoing the surgery remains at approximately 4%, especially in *BRCA1* mutation carriers [6,8,24,35]. The incidence of occult cancer in *BRCA1/2* mutation carriers at the time of risk-reducing surgery is 2.5 to 3.6% [4,6].

Given the high level of effectiveness in primary prevention of ovarian cancer, the potential efficacy in preventing breast cancer, and an increased overall reduction in mortality, RRSO is highly recommended for women carrying the *BRCA1/2* mutations, according to the recommendations of the NCCN and a joint position statement of the Society of Gynecologic Oncology and the American College of Obstetricians and Gynecologists [1,2,3,4,6,7,8,24,25,26,27,29,32]. However, the removal of apparently healthy organs has physiological implications, with concomitant sexual and psychological impacts.

### 2.3. Physiological Implications of RRSO

Despite being considered a safe surgical procedure [6], RRSO has several physiological implications, mainly related to the surgical removal of the ovaries. Indeed, RRSO will lead to a surgical menopause, associated with a sudden decrease in estrogen levels and, consequently, with the onset of menopausal distress, including vasomotor symptoms, genitourinary syndrome, sleep disturbances, mood swings, and sexual dysfunction (e.g., decreased libido, vaginal dryness, and dyspareunia). These symptoms are generally more severe than in natural, gradual menopause [1,3,7,25].

Bilateral RRSO has also been associated with an increased non-cancer-related morbidity, such as increased risk of osteoporosis, cardiovascular disease, and metabolic syndrome, even though further studies are warranted [1,4,6,25]. First, bone mineral density decreases by up to 6.7% in premenopausal women at 12 months after an oophorectomy, which is much higher than the rate observed in natural menopause [39]. Second, despite the absence of prospective data, cohort studies show a slightly increased risk of cardiovascular disease in premenopausal women undergoing RRSO [40]. Third, these women may experience negative changes in lipid profile, with subsequent development of atherosclerosis [41].

Additionally, the recommendation to offer a RRSO from the age of 35 also limits the fertility window and could represent a major concern, especially in developed countries where the mean age of the first pregnancy is postponed [42].

Recently, and considering the existing evidence that many ovarian cancers originate in the fallopian tubes, it has been suggested that a risk-reducing salpingectomy alone, or followed by an oophorectomy close to the age of natural menopause, might postpone the onset of early menopause symptoms and allow an extended fertility window. However, the level of risk reduction achieved through this strategy is unknown, and data regarding the efficacy of this approach are lacking [1,6,7,8,27,36].

Hormone replacement therapy (HRT) (i.e., the exogenous administration of estrogens) has been recommended to women without a personal history of breast cancer, in the absence of counterindications, and until the natural age of menopause, in order to reduce menopausal distress following RRSO [1,2,8,25,29]. According to a systematic review evaluating the risks and benefits of HRT, this therapy was associated with improved quality of life, better sexual functioning and bone health, less menopausal distress, and reduced risk of cardiovascular disease after RRSO [1,2,3,6,7,25]. Although concerns have been raised about a possible increase in risk of breast cancer with the use of HRT, a large meta-analysis of 1100 women with pathogenic *BRCA1/2* variants undergoing RRSO found no increased risk of breast cancer with short-term use of HRT (HR 0.98; 95% CI (0.63–1.52)) [1]. Nevertheless, large, controlled trials are urgently needed, and women should be informed that the existing data are limited [7,8,25].

## 3. The Psychological Process of Deciding to Undergo RRSO

The decision-making process to undergo RRSO is fraught with uncertainty and complexity for women carrying *BRCA1/2* mutations [13,21].

Several factors have been described in the literature as affecting the woman’s decision to undergo, delay, or avoid this surgical intervention [12]. Having a family or personal history of cancer, or having witnessed a close relative dying of cancer, are commonly reported reasons for women favorably choosing RRSO (e.g., [14,43,44]). Demographic factors, such as women’s age and parity, have also been examined in relation to the choice to undergo risk-reducing interventions, suggesting that women closer to menopausal age and with children are more likely to opt for this prophylactic measure [43,45].

It is worth noting that psychological factors have often been reported by women as variables that may hinder the decision-making process, including worries about getting cancer [46], or about the physiological, emotional, and sexual consequences of ovary removal surgery, and surgical menopause [44]. In addition, the availability of internal (e.g., coping skills, self-efficacy) and external (e.g., social support) resources to deal with post-surgery physical and emotional consequences has been commented upon as a key psychological determinant underlying the decision to undergo RRSO [13]. However, most of these studies relied on small samples and qualitative designs, often lacking a solid theoretical framework to guide the research into this complex decision-making process. Besides the methodological deficiencies, the absence of a consistent theoretical model is particularly noteworthy. First, a theoretical model might be used to guide research in a theory-driven manner, permitting, for instance, consistency of results, conceptual coherence, or comparability between results. Second, it might also impact clinical practice by facilitating the physician’s case conceptualization in clinical shared decision-making.

**Figure 1:**
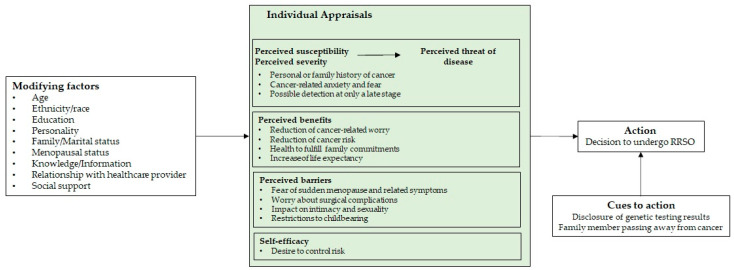
The adapted application of the HBM to women’s decision to undergo RRSO. Key concepts: Perceived susceptibility—belief about the likelihood of getting a disease or condition; Perceived severity—belief about the seriousness of contracting an illness or of leaving it untreated; Perceived threat of disease—combination of perceived susceptibility and severity; Perceived benefits—belief in efficacy of advised action to reduce risk; Perceived barriers—belief about the tangible and psychological costs of the advised action; Self-efficacy—confidence in one’s ability to take action. How to read the model: when confronted with contextual cues that demand a decision as to whether to undergo RRSO, women are more likely to decide favorably if they regard themselves as susceptible to cancer, believe that getting cancer would have potentially serious consequences, believe that the benefits of undergoing RRSO outweigh its costs (barriers), and believe they are capable of making that decision. All these appraisals may also be influenced by women’s sociodemographic, psychological, structural, and relational factors (adapted from [49,50]).

Recently, some researchers applied the Health Belief Model (HBM) [47,48] to examine the decision-making process of women who choose a RRSO. The HBM was developed to explain health behavior change, and portrays a complex interplay of intrapersonal factors, while taking in consideration the context in which health behaviors take place [49,50]. According to this model, when confronted with environmental health-related cues to action, a person’s behavior is guided by their internal appraisals of the perceived threat of a disease (e.g., beliefs about getting a disease and its seriousness); the perceived benefits and barriers of adopting a given health behavior; and the perceived self-efficacy in maintaining such behavior. Additionally, these individual perceptions may vary according to individual attributes, such as age, education, or knowledge [49]. The adaptation of the HBM to women’s decision to undergo RRSO is depicted in Figure 1, based upon currently existing models [51,52].

### 3.1. The Psychosocial Impact of Undergoing RRSO

As for the psychological impact of having the surgery per se, the available studies report mixed results. On the one hand, some researchers have found the levels of overall post-surgery quality of life to be comparable to those of the general population, along with a significant decrease in cancer-related worry (e.g., [53,54,55]). On the other hand, there is also evidence for impaired sexual functioning, persistent physiological symptoms derived from surgical menopause and hormonal changes (e.g., vaginal dryness, hot flashes, decreased libido), and reduced body image satisfaction in women who underwent RRSO [20,31,56]. Moreover, it has been reported that around 20% of these women continue to present cancer-specific distress after surgery [53,57,58], and increased depressive and anxiety symptoms that tend to persist during one-year post-surgery [59]. Considering the holistic nature of potential consequences of RRSO (i.e., physical, psychological, relational, social), a multidisciplinary approach would be desirable to manage the deleterious effects of the surgery and support *BRCA* carriers in their decision-making process and post-surgery adaptation. However, even though clinical guidelines often recommend this approach, it is not always reflected in clinical practice, due to economic and organizational factors, which might contribute to the pervasiveness of the negative consequences of surgical menopause [6,8,26,27,28].

Undergoing RRSO surgery tends to be emotionally challenging, with serious physical and psychosocial consequences that may conflict with its primary purpose, and thus impair the decision-making process for women who carry a *BRCA* mutation. However, little is known about the role of modifiable psychosocial mechanisms that seem to shape the decision-making process, and women’s subsequent adaptation to its outcomes [14,45]. Notably, physician-patient communication has been increasingly acknowledged as a crucial modifiable variable that appears to be uniquely linked to women’s adaptation outcomes, following their decision to (not) undergo RRSO (e.g., [13,19,20,21]).

### 3.2. The Role of Physician-Patient Communication in the Decision-Making Process and in Post-Surgery Adaptation

Shared decision-making (SDM) consists of joint participation from both physician and patient in making a health decision, through the discussion of the available options and their benefits and harms, while considering the patient’s values, preferences, and circumstances [60]. There are several SDM models used in healthcare practice [61], with some of them specifically developed for oncology care (e.g., [62,63]). Even though no specific model has yet been examined in the context of the decision to undergo prophylactic surgery in mutation carriers, it is possible to establish a connection between the SDM model suggested by Shay and Lafata (2014) and the previously described HBM model. According to this SDM model, heavily drawn from the patients’ perspective, making a health decision depends on the interaction of several factors, such as: patient self-advocacy; open-mindedness and mutual respect between physician and patient; a physician’s personalized recommendation; and mutual information exchange. These factors occur in the context of the relationship and communication between physician and patient [64]. Considering the aforementioned HBM model, physician-patient communication may, therefore, be considered one of the modifying factors that uniquely shapes the women’s subjective appraisals that lead them to make the decision for RRSO.

Physician-patient communication (PPC), and particularly physicians’ empathic communication skills, are at the core of patient-centered healthcare and of medical practice [65,66]. Research has highlighted the positive effects of PPC on patients’ physical and emotional health improvements (e.g., [16,67]), treatment adherence [17,68], and overall well-being [18,69] across several healthcare settings, including cancer care [70].

In the context of decision-making for RRSO in women carrying a *BRCA* mutation, there are a few studies where the impact of PPC on women’s adjustment was examined. Some qualitative studies commented that even though women were frequently satisfied with their surgery decision, there was also dissatisfaction with the information provided by their physicians before the surgery, which could ultimately worsen the women’s ability to cope with deleterious post-surgery effects. Typically, these clinical information topics relate to possible treatment alternatives, negative impacts of menopausal symptoms, or the potential changes on sexuality and sexual functioning [21,56,71,72]. In a recent qualitative study describing the psychological experiences of women who underwent RRSO, it was found that, in the majority of cases, physicians failed to provide complete information, showed low levels of empathy and respect for the women’s values, preferences, and individuality, and adopted a disease-focused posture (i.e., focusing on organ health or exclusive disease prevention, and disregarding organism-context interactions and health promotion). Following these clinical attitudes, women experienced an increased psychological burden in the decision-making process, sought other professionals, or unwillingly delayed the surgery [14].

Moreover, some researchers have recently begun to analyze, using a quantitative approach, the impact of PPC on the psychological adjustment outcomes of having a RRSO. For instance, Zarbo and colleagues (2022) suggested that a greater satisfaction with medical communication could be associated with the provision of accurate and complete information about risk, prevention strategies, and post-surgery effects, while using an empathic and patient-centered style. In turn, satisfaction with medical communication may enhance patients’ self-efficacy, cultivate positive perceptions of agency, and contribute to decreased cancer-related anxiety. Furthermore, women who underwent RRSO and experienced greater satisfaction with medical communication reported reduced levels of cancer anxiety and experienced greater levels of post-surgery psychological quality of life [73].

## 4. Recommendations to Improve Clinical Practice

This review has shown that premenopausal women carrying a *BRCA1*/*2* mutation, with an increased risk of developing ovarian or breast cancer, face a challenging process when deciding to undergo a RRSO. The available studies indicate that the physicians’ ability to sensitively inform, guide, and support women in that process is related to improved post-surgery health outcomes. Even if not abundant and marked by several methodological limitations, the existing research enables the outlining of some recommendations for physicians to improve their practice in this healthcare context, in addition to the already existing clinical guidelines.

First, some harmful impacts of the physiological and psychological consequences of undergoing an RSSO may be reduced, or even prevented, through sensitive physician-patient communication. More often than not, research has shown that physicians’ poor-quality communication competencies negatively impact women’s psychological adaptation during the decision-making process and after RRSO surgery. Therefore, training and developing physicians’ communication skills should be emphasized in medical curricula and continuing professional development [21,73]. Some communication skills training programs for physicians have shown promising results in the improvement of empathy, active listening, non-verbal communication, and emotion regulation (e.g., Empathetics) [74,75]. In addition, recently, mindfulness-based approaches have shown their feasibility within communication skills training for physicians, possibly enhancing adaptive emotion regulation (in self and others), psychological flexibility, and compassion [65].

Second, a constant update of evidence-based clinical guidelines is vital in order to provide the best care possible, while remaining mindful of the potentially enduring negative consequences of this procedure and providing the best information and recommendations to every woman. The combination of advanced technical competence and advanced interpersonal skills is consistent with the values underlying shared decision-making and patient-centered care, leading to patients’ higher satisfaction with healthcare and improved adaptation outcomes.

Third, given the inherent physical and psychosocial impacts of RRSO, it has been suggested that counselling in this context should be interdisciplinary [6,14,71]. A multidisciplinary team encompassing physicians/oncologists, nurses, clinical psychologists, physical therapists, and social workers is likely to enable the comprehensive assessment and management of these women’s complex healthcare needs. Additionally, due to the potentially negative impact of RRSO on intimate relationships, it has been suggested that counselling should be extended to family relatives, particularly the spouse/partner [19]. Specifically, women have frequently reported that sexuality and intimacy tend to be overlooked by health professionals in the discussion of the impact of RRSO [13]. Bearing in mind that sexual health is part of overall well-being and quality of life [76], physicians should be cognizant of the importance of addressing that health dimension in the context of the shared decision-making process related to RRSO [19,20,71].

## 5. Future Research Directions

Hereditary risk for the development of breast or ovarian cancer, due to *BRCA1* or *2* mutations, along with the respective prophylactic measures, require further investigation, in order to improve the understanding of best clinical practices.

There is a reiterated need to deepen the research into breast and ovarian cancer and, particularly, into technological means and imaging techniques for early screening and detection [77,78]. Currently, risk-reducing interventions, such as RRSO, call for more efficacy studies to increase physicians’ and patients’ confidence in this procedure. In this case, a call for research is particularly directed to prophylactic interventions, namely early salpingectomy with delayed oophorectomy, which may postpone the onset of early menopause symptoms and allow an extended fertility window, thus buffering the deleterious effects of RRSO on psychosocial outcomes and increasing the chances for women’s improved quality of life [11]. Additionally, considering the broad scope of hereditary genomic mutations, less prevalent than *BRCA* but still conferring an increased risk to develop breast and/or ovarian cancer, future research should also focus on the psychological challenges faced by non-*BRCA* predisposing mutation carriers to better inform the establishment of clinical management protocols.

This review also revealed the need for further research about the psychosocial effects and modifiable mechanisms involved in women’s decision-making process and post-surgery adjustment, while adopting multi-method and longitudinal designs. Specifically, an investment in quantitative research into the role and impact of PPC is necessary, given its unique links to women’s decision-making processes and adjustment outcomes. Moreover, quantitative studies, complementary to qualitative research, should provide more robust and less biased evidence about the mechanisms through which PPC impacts decision-making and adjustment outcomes, thus allowing for a more comprehensive empirical framework for clinical intervention and the improvement of clinical guidelines.

## Figures and Tables

**Table 1:** Hereditary genomic mutation risk rates for developing breast and/or ovarian cancer.

Gene	Ovarian Cancer Cumulative Risk (by Age ≥ 70)	Breast Cancer Cumulative Risk (by Age ≥ 70)
*MLH1*	4–20%	Unknown
*MSH2*	8–38%	Unknown
*MSH6*	1–13%	Unknown
*PALB2*	Unknown	35%
*ATM*	Unknown	33%
*STK11*	18–21%	45–50%
*BRIP1*	7–10%	Unknown
*RAD51C/D*	5–12%	Unknown
*BRCA1*	45–60%	65–80%
*BRCA2*	11–35%	50–70%

**Table 2:** Risk-reducing measures recommended by clinical guidelines for *BRCA* mutation carriers.

Risk-Reducing Measure	Recommended Implementation
Screening	
Breast examinations (mammography, MRI)	>age 25
Pelvic ultrasound testing + serum biomarkers	>age 35
Risk-reducing agents	
Oral contraceptives	More data needed
Chemoprevention	More data needed
Risk-reducing surgeries	
Mastectomy (RRM)	No age defined
Salpingo-oophorectomy (RRSO)	>age 35–40 (*BRCA1*) >age 40–45 (*BRCA2*)
